# Supplementary material for: Splice-Junction-Based Mapping of Alternative Isoforms in the Human Proteome
Source: Cell Rep. Author manuscript; Available in PMC 2020 Jan 15. (PMC6961840; doi:10.1016/j.celrep.2019.11.026)
Supplement: 2 [file NIHMS1546469-supplement-2.zip › DF1/PXD006675/LeftAtrium/LeftAtrium_13_TTN_RVEAEPAEEVTIMEEK.pdf]

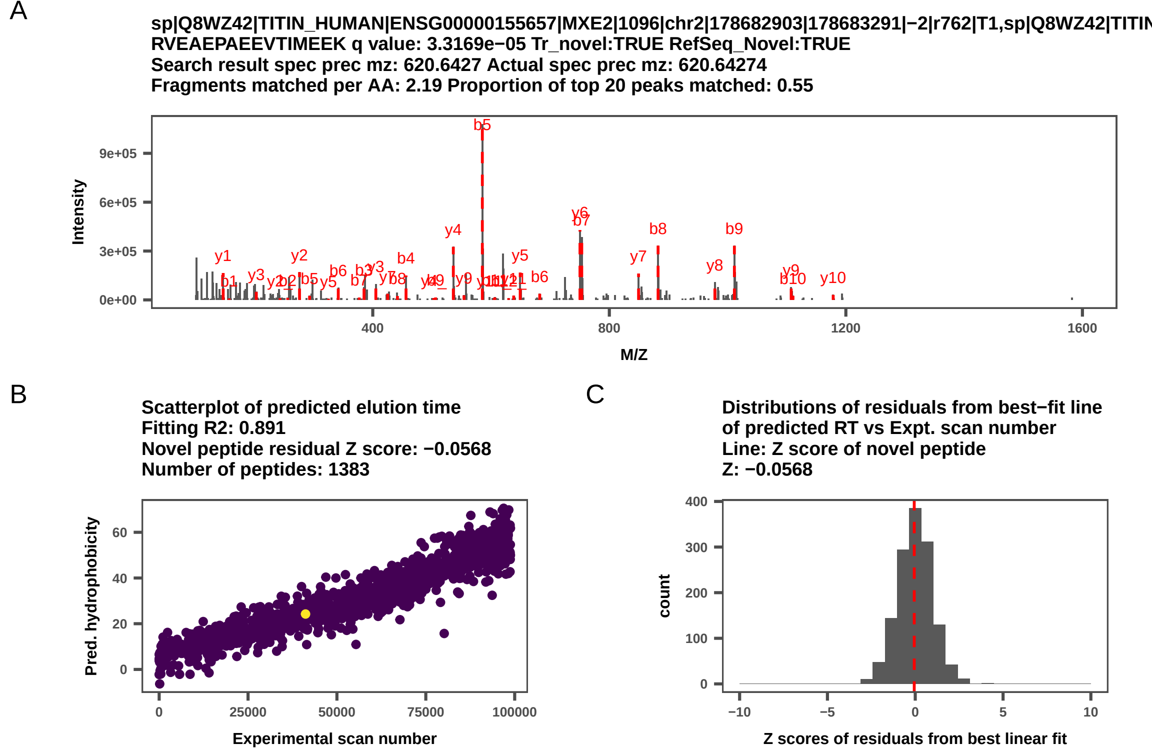
A
sp|Q8WZ42|TITIN_HUMAN|ENSG00000155657|MXE2|1096|chr2|178682903|178683291|−2|r762|T1,sp|Q8WZ42|TITIN
RVEAEPAEEVTIMEEK q value: 3.3169e−05 Tr_novel:TRUE RefSeq_Novel:TRUE
Search result spec prec mz: 620.6427 Actual spec prec mz: 620.64274
Fragments matched per AA: 2.19 Proportion of top 20 peaks matched: 0.55
Intensity
9e+05
6e+05
3e+05
0e+00
b5
y6
b7
y4
b8
b9
y1
y2
y3
b4
y5
y7
y8
y9
b10
y10
b6
M/Z
400
800
1200
1600
B
Scatterplot of predicted elution time
Fitting R2: 0.891
Novel peptide residual Z score: −0.0568
Number of peptides: 1383
Pred. hydrophobicity
60
40
20
0
0
25000
50000
75000
100000
Experimental scan number
C
Distributions of residuals from best−fit line
of predicted RT vs Expt. scan number
Line: Z score of novel peptide
Z: −0.0568
count
400
300
200
100
0
−10
−5
0
5
10
Z scores of residuals from best linear fit
